# Body mass regulation in humans is associated with dopamine synthesis capacity and dopamine receptor profile measured with positron emission tomography

**DOI:** 10.1038/s41398-026-04318-6

**Published:** 2026-08-13

**Authors:** Angela M. Ianni, Catherine E. Hegarty, Joseph C. Masdeu, Michael D. Gregory, Philip D. Kohn, Daniel P. Eisenberg, Karen F. Berman

**Affiliations:** 1 Clinical & Translational Neuroscience Branch, National Institutes of Mental Health, Intramural Research Program, National Institutes of Health, Bethesda, MD USA; 2 Present Address: Department of Psychiatry, University of Pittsburgh, Pittsburgh, PA USA; 3 Present Address: Department of Neurology, Nantz National Alzheimer Center, Houston Methodist and Weill Cornell Medicine, Houston Methodist, Houston, TX USA

**Keywords:** Molecular neuroscience, Physiology

## Abstract

The widespread and increasing prevalence of excess body mass represents a major public health concern and has been hypothesized to involve dysfunction of the dopaminergic reward system. However, a detailed, systems-level understanding of interactions between the dopamine system and body mass regulation in humans has been elusive. To address this knowledge gap, we employed positron emission tomography (PET) to directly measure presynaptic dopamine synthesis capacity (with [^18^F]-FDOPA) and dopamine receptor availability (D_1_ and D_2/3_ receptors with [^11^C]-NNC112 and [^18^F]-Fallypride, respectively) in 117 individuals with body mass index (BMI) values ranging from normal to moderately obese. We found that elevated BMI was associated with heightened dopamine synthesis capacity in the midbrain and hypothalamus, key regions for homeostatic appetite control, as well as with lower D_2/3_ receptor availability in the midbrain, where D_2_ receptors play an important role in the autoregulation of striatal dopamine release. Moreover, we found that the presence and rate of weight gain in the 1–2 years prior to scanning was associated with increased presynaptic dopamine synthesis capacity in the ventral striatum and midbrain, important components of the reward network. These data suggest that upregulation of body mass beyond the normal range is related to increased dopamine synthesis capacity in reward- and homeostatic-related regions coupled with blunted autoregulation.

## Introduction

Over 40% of adults worldwide and 70% in developed countries such as the United States are overweight, with prevalence more than doubling in the past 30 years [1, 2]. It is clear from extensive prior scientific work that activity and consumption behaviors underlying body mass adaptations rely on homeostatic and motivational neurocircuitry [3]. The regulation of eating behaviors, for instance, involves a number of factors including peripheral signals that are transported to the brain (e.g., ghrelin, leptin, insulin), energy homeostatic mechanisms occurring in the hypothalamus, and function of the reward system [3]. Dopaminergic mechanisms are thought to play a role in all of these factors including interactions with peripheral signals [4–7], regulation of appetitive neurotransmission in the hypothalamus [8–10], and, most prominently, a central role of the mesolimbic reward system (i.e., the ventral tegmental area of the midbrain and its projections, including the ventral striatum) [11–13]. Accordingly, substantial evidence has suggested a direct link between dopamine and weight control, with the mesolimbic system being most frequently implicated in human studies [3, 9, 14–16].

Abnormalities of the mesolimbic dopamine system have been implicated in both the development and progression of obesity [17, 18]. Studies directly measuring dopamine receptor availability in vivo using positron emission tomography (PET) are central to this research area (for a comprehensive review, see Janssen et al. [19]). However, findings about the role of D_2/3_ receptors in obesity have been inconsistent, particularly among individuals with mild to moderate obesity. A seminal study by Wang et al. reported decreased striatal dopamine D_2/3_ receptor levels in people with morbid obesity [17], a result replicated in a subsequent human PET study [20] and rodent models, where D_2/3_ receptor availability correlated with future weight gain and food seeking-behavior [18]. Yet, other studies have failed to replicate this finding [21–26], reported opposite effects [27–30], or identified complex regional patterns—for example, a negative correlation between BMI and D_2/3_ receptor binding potential in the ventromedial striatum but a positive correlation in the dorsolateral striatum [31]. More recently, a controlled inpatient study employing both high- and low-affinity dopamine D_2/3_ receptor tracers in adults spanning a wide BMI range suggested that adiposity relates more to increased striatal dopamine tone than to differences in receptor availability [32]. In contrast, studies investigating the role of dopamine synthesis capacity—a proxy for dopamine tone—have generally found negative correlations with BMI, although some studies focused exclusively on the striatum had limited sample sizes [33–35]. Finally, while animal research implicates dopamine D_1_ receptors in appetite and weight regulation [36], human studies of this important component of the dopamine system are lacking.

These inconsistencies in the evidence delineating the role of dopamine in the accumulation of excess body mass have led to competing theories about the primacy of dopaminergic abnormalities in obesity. For instance, both dopaminergic hypofunction (‘reward-deficiency’) [37–39] and dopaminergic hyperfunction (‘reward surfeit’) [40, 41] models have been proposed, as well as a model of imbalance between motivational and inhibitory circuits [14, 42]. Moreover, only select aspects of the multifaceted dopamine system have been explored thus far, and the lack of an integrated understanding of particular dopaminergic system characteristics in relation to states of body mass maintenance remains a barrier to better insight into the neural mechanisms most relevant to the obesity epidemic and to development of targeted, personalized pharmacological treatments.

To address this knowledge gap, we used multimodal PET imaging to study a cohort of 117 healthy humans in the normal to obese BMI range and assessed not only D_2/3_ receptor availability, but also presynaptic dopamine synthesis capacity and D_1_ receptor availability in the striatum, midbrain, and hypothalamus, key regions in the reward and homeostatic networks. We tested the hypothesis that increased body mass is associated with a model of diminished dopamine receptors and enhanced presynaptic dopaminergic tone.

We sought to clarify previous findings (1) with a relatively large sample size spanning the normal to obese range, (2) by including in our analysis regions important for appetite and reward regulation but not previously tested in human PET studies (midbrain and hypothalamus), and (3) by also assessing the dopaminergic signature of prior weight gain. In particular, we sought to add clarity to the mixed literature on the role of dopamine D_2/3_ receptors in excess body mass, investigating whether results in previous human and rodent studies showing a link between obesity per se and diminished dopamine D_2/3_ receptors [17, 18] would be parametrically observed in our large sample of people with lower obesity severity. Furthermore, whether these prior findings that have been generally observed in the setting of severe obesity extend to the more modest levels of excess body mass is not clear.

## Methods

### Participants

One-hundred-seventeen healthy adult volunteers (103 right-handed, 6 left-handed, 7 ambidextrous, and 1 unknown) with BMIs ranging from normal to moderately obese were recruited from the local community and screened by physician-administered physical/neurological examination, standardized clinical interview (SCID) [43], laboratory tests, and structural MRI read by a radiologist to rule out psychiatric, neurological, and major medical illness. Participants were not taking medications that interfere with central dopaminergic signaling (e.g., dopamine agonists or antagonists) at the time of scans. Of the 117 participants, 115 completed the [^18^F]-FDOPA presynaptic dopamine synthesis capacity PET scan, 76 completed the [^11^C]-NNC112 D_1_ receptor scan, and 72 completed the [^18^F]-Fallypride D_2/3_ receptor scan (Table 1). All participants provided informed consent and were compensated for their participation. Study procedures were approved by the NIH Institutional Review Board and NIH Radiation Safety Committee.

**Table 1:** Participant Demographics.

Analysis	Number of subjects	Sex (F/M)	Age (mean ± std dev)	BMI range (mean ± std dev)
All	117	60/57	34.3 ± 8.9	18.3–36.1 (25.1 ± 3.7)
**Current BMI**				
[^18^F]-FDOPA	115	58/57	32.9 ± 8.5	18.5–34.8 (25.1 ± 3.7)
[^18^F]-Fallypride	72	36/36	35.4 ± 9.2	19.7–36.1 (25.2 ± 3.7)
[^11^C]-NNC112	76	41/35	34.8 ± 9.0	18.3–35.9 (25.0 ± 3.8)
**Prior BMI change**				
[^18^F]-FDOPA	Total: 59 Gained weight: 33 Lost weight: 26	32/27	32.5 ± 8.3	
[^18^F]-Fallypride	Total: 42 Gained weight: 22 Lost weight: 20	25/17	34.6 ± 8.2	
[^11^C]-NNC112	Total: 42 Gained weight: 21 Lost weight: 21	25/17	34.1 ± 7.6	

### BMI calculations

Body mass index (BMI) was calculated using height and weight measurements recorded by nursing staff around the time of the PET scan (1.6 ± 10.4 [mean ± SD] weeks prior to scan). In addition, for a subset of the cohort, BMI was also calculated 1–2 years prior to the PET scan (for [^18^F]-FDOPA: 59 participants, mean prior BMI measurement date 1.43 ± 0.24 years before PET scan; for [^11^C]-NNC112: 42 participants, mean prior BMI measurement 1.51 ± 0.22 years before PET scan; [^18^F]-Fallypride: 42 participants, mean prior BMI measurement 1.49 ± 0.25 years before PET scan). Analyses of BMI change were conducted two ways: (1) participants were categorized into weight-gain or -loss groups based on their change in BMI between the prior value and the value at the time of the PET scan, and (2) the rate of BMI change for each participant was calculated by dividing this difference by the time elapsed between the two measurements and analyzed parametrically.

### PET data acquisition

On separate days, each participant completed three positron emission tomography (PET) scans to assess presynaptic dopamine synthesis capacity (with [^18^F]-FDOPA), and D_1_ and D_2/3_ receptor binding potential (with [^11^C]-NNC112 and [^18^F]-Fallypride, respectively). PET data were collected on a GE Advance 3D PET scanner (for all [^18^F]-FDOPA scans) and a Siemens HRRT scanner (for all [^18^F]-Fallypride and [^11^C]-NNC112 scans). Following an eight-minute transmission scan, dynamically binned emission scans were collected for 90 min after IV tracer injection (for [^18^F]-FDOPA and [^11^C]-NNC112) measurements, and for [^18^F]-Fallypride measurements for four hours after tracer injection. The tracer dose administered was 15.82 ± 1.21 mCi for [^18^F]-FDOPA, 5.11 ± 0.12 mCi for [^18^F]-Fallypride, and 19.52 ± 0.59 mCi for [^11^C]-NNC112. Caffeine and nicotine were restricted for four hours prior to the scans and food for six hours preceding the scan (for [^18^F]-FDOPA only, to reduce competition for transport of the tracer into the brain). Also, for the [^18^F]-FDOPA scans, participants were pretreated with carbidopa 200 mg PO one hour prior to injection to reduce peripheral degradation of the tracer. All PET scans were conducted in the Eastern Time zone during a standard window between approximately 8:30 and 14:30. Participants also completed a T1-weighted magnetic resonance imaging (MRI) scan which was used for registration and brain segmentation.

### PET analyses

PET data underwent reconstruction with attenuation correction and were motion-corrected with alignment of all timepoints. We then performed both native-space region of interest (ROI) analyses of subregions within the basal ganglia as well as voxelwise standard-space analyses to search for localized results within our predefined networks of interest: the basal ganglia (reward network) and the hypothalamus (homeostatic network). For both methods, we used non-invasive reference input models for PET parameter modeling, implemented with the PMOD software (http://www.pmod.com/web/). The [^18^F]-FDOPA uptake rate (K_i_) was calculated with the Gjedde-Patlak method [44, 45], while [^11^C]-NNC112 and [^18^F]-Fallypride binding potentials (BP_ND_) were calculated with the SRTM method [46]. A centroinferior gray matter region of the cerebellum, excluding the vermis to minimize specific binding and omitting the lateral and superior parasinus regions, was used as the reference region for modeling all ligands.

For ROI analyses, native-space T1-weighted MRI images were corrected for intensity nonuniformity [47] and aligned to the anterior-posterior commissure. Freesurfer (http://freesurfer.net/) was used for initial segmentation of bilateral subregions of the basal ganglia, where dopamine receptors are most abundant and where past work has implicated dopaminergic system alterations in obesity: bilateral dorsal putamen, dorsal caudate nucleus, ventral striatum, and dopaminergic midbrain. ROIs were manually refined by raters blinded to the PET data to ensure accurate segmentation and were eroded by one voxel to minimize partial volume effects. MRI scans were registered to native space PET images, and values were extracted for each of the ROIs. Midbrain data for the D_1_ receptor tracer ([^11^C]-NNC112) were excluded due to low signal reliability in this region, as indicated by poor fit of the model. For each PET tracer, we conducted linear regression analyses using either current BMI or rate of BMI change as the independent variable, with ROI values as the dependent variables. All variables were standardized, and models included age and sex as covariates. Descriptive statistics for all PET imaging outcome measures ([^18^F]-FDOPA K_i_, [^18^F]-Fallypride BP_ND_, and [^11^C]-NNC112 BP_ND_) across regions of interest are provided in eTable 1. Cross-correlations of ROI-level PET values across tracers were additionally computed to assess inter-tracer associations within the 63 subjects who completed scans with all three radiotracers (eFig. 1). Analyses were performed in R (v4.3.1) using the *lm* function [48]. Group comparisons of PET values between individuals who gained versus lost weight were conducted using analysis of variance via the R *aov* function [48]. For linear regressions that included a single model per PET tracer, significance was assessed using a threshold of *p* < 0.05. For ANOVA analyses that required separate models for each ROI, significance was assessed using Bonferroni correction for multiple comparisons across all ROIs (four for [^18^F]-FDOPA and [^18^F]-Fallypride, three for [^11^C]-NNC112) [49] (see Table 2). Power analyses for multiple regression conducted in R using the pwr package [50] (*α* = 0.05, power = 0.80) indicated that sample sizes were adequately powered to detect medium-to-large effect sizes (i.e., f^2^ ≥ 0.25) for ROI analyses.

**Table 2:** Region of Interest Results.

Current BMI						
PET tracer	Contrast	Model-fit indices	Region	Coefficient	*p*-value	Cohen’s f^2^ (partial)
[^18^F]-FDOPA	Association with Current BMI	F = 1.43, *p* = 0.21, df=108, R^2^ = 0.07, adj. R^2^ = 0.02, Cohen’s f^2^ (global) = 0.08	Putamen	−0.15	0.32	<0.01
			Caudate nucleus	−0.05	0.75	<0.01
			Ventral striatum	0.13	0.27	0.03
			Midbrain	0.16	0.15	0.02
[^11^C]-NNC112	Association with Current BMI	F = 1.11, *p* = 0.36, df = 70, R^2^ = 0.07, adj.R^2^ = 0.01, Cohen’s f^2^ (global) = 0.08	Putamen	0.21	0.40	<0.01
			Caudate nucleus	−0.25	0.39	0.02
			Ventral striatum	0.01	0.95	<0.01
[^18^F]-Fallypride	Association with Current BMI	**F** = **2.67, p** = **0.02, df** = **55,** **R**^**2**^ = **0.23, adj. R**^**2**^ = **0.14,** **Cohen’s f**^2^ **(global)** = **0.29**	Putamen	0.29	0.34	<0.01
			Caudate nucleus	0.20	0.59	0.02
			Ventral striatum	−0.46	0.07	0.07
			**Midbrain**	−**0.34**	**0.03**	**0.13**

BMI Change Group						
PET tracer	Contrast	Region	F-statistic	*p*-value (uncorrected)	Bonferroni-corrected *p*-value	Cohen’s f (partial)
[^18^F]-FDOPA	Weight gain vs. Weight loss subjects	Putamen	0.04	0.84	3.35	0.03
		Caudate nucleus	0.23	0.64	2.54	0.06
		**Ventral striatum**	**6.89**	**0.01**	**0.04**	**0.35**
		Midbrain	0.12	0.73	2.93	0.05
[^11^C]-NNC112	Weight gain vs. Weight loss subjects	Putamen	0.42	0.52	1.57	0.10
		Caudate nucleus	0.01	0.91	2.74	0.02
		Ventral striatum	0.41	0.53	1.58	0.10
[^18^F]-Fallypride	Weight gain vs. Weight loss subjects	Putamen	<0.01	0.98	3.90	<0.01
		Caudate nucleus	0.21	0.65	2.61	0.07
		Ventral striatum	<0.01	0.94	3.76	0.01
		Midbrain	0.16	0.70	2.78	0.07

Rate of BMI change						
PET tracer	Contrast	Model-fit indices	Region	Coefficient	*p*-value	Cohen’s f^2^ (partial)
[^18^F]-FDOPA	Association with Rate of BMI change	F = 1.47, *p* = 0.207, df = 52, R^2^ = 0.14, adj. R^2^ = 0.05, Cohen’s f^2^ (global) = 0.17	Putamen	0.49	0.63	<0.01
			Caudate nucleus	−1.26	0.21	0.01
			**Ventral striatum**	**2.04**	**0.05**	**0.08**
			Midbrain	−0.89	0.38	0.02
[^11^C]-NNC112	Association with Rate of BMI change	F = 0.29, *p* = 0.916, df = 36, R^2^ = 0.04, adj. R^2^ = −0.10, Cohen’s f^2^ (global) = 0.04	Putamen	0.42	0.68	<0.01
			Caudate nucleus	0.32	0.76	<0.01
			Ventral striatum	−0.49	0.63	0.01
[^18^F]-Fallypride	Association with Rate of BMI change	F = 0.43, *p* = 0.854, df = 30, R^2^ = 0.08, adj. R^2^ = −0.11, Cohen’s f^2^ (global) = 0.09	Putamen	0.59	0.56	<0.01
			Caudate nucleus	−1.41	0.17	0.01
			Ventral striatum	1.28	0.21	0.05
			Midbrain	0.44	0.66	<0.01

For voxel-wise analyses, PET scans were anatomically normalized to Montreal Neurological Institutes (MNI) standard space and smoothed with a 10 mm^3^ kernel before undergoing modeling in PMOD. FSL [51] was used to implement voxel-wise general linear model analyses to test for correlations with BMI and for differences between BMI change groups, controlling for age and sex. Statistical significance was assessed using nonparametric permutation inference, implemented with FSL’s randomise tool [52]. Specifically, 10,000 permutations were run to generate the null distribution and the threshold-free cluster enhancement (TFCE) test statistic was calculated [53]. Significance was assessed using a statistical threshold of *p* < 0.05, FWE-corrected for multiple comparisons within a single ROI that included the basal ganglia and hypothalamus for small volume correction (Table 3, “TFCE-corrected *p*-value (FWE)”). The basal ganglia was defined using a similar method to the native-space ROI creation, except performing the Freesurfer segmentation on an average T1-weighted MRI created from 240 healthy volunteers and including regions from the striatum and dopaminergic midbrain. The hypothalamus region was created using the WFU PickAtlas software package (Wake Forest University, Winston-Salem, NC, USA) and dilated volumetrically by 3 mm. [^18^F]-FDOPA data from one participant were excluded due to poor quality. Midbrain ROI data for [^18^F]-Fallypride were excluded from ten participants due to inadequate SRTM model fit.

**Table 3:** Voxel-wise Results^a^.

Contrast	Brain region	Peak coordinate (MNI) (x, y, z)	TFCE-corrected *p*-value (FWE)	Z-statistic	Cluster size (voxels)
[^18^F]-DOPA K_i_ Positive Correlation with Current BMI	Left midbrain	(−10.5, −21, −21)	0.025	3.60	183
[^18^F]-DOPA K_i_ Positive Correlation with Current BMI	Right hypothalamus	(9, 0, −16.5)	0.026	3.99	69
[^18^F]-DOPA K_i_: Weight gain > Weight loss subjects	Right ventral striatum	(21, 6, −13.5)	0.001	4.40	756
[^18^F]-DOPA K_i_: Weight gain > Weight loss subjects	Left ventral striatum	(−21, 13.5, −12)	0.028	3.12	397
[^18^F]-DOPA K_i_: Weight gain > Weight loss subjects	Left midbrain	(−1.5, −30, −24)	0.015	3.92	228

^a^Search volume restricted to single ROI comprised of the basal ganglia and hypothalamus.

## Results

### Demographics

One-hundred-seventeen healthy volunteers aged 19 to 52 years (mean 34.3 ± 8.9, 60 females) with body mass indices ranging from 18.3 to 36.1 (mean 25.1 ± 3.7) participated in this study. Demographic information for the participants who completed each PET scan is shown in Table 1, and effects across demographic measures are reported below with liberal, uncorrected *p*-values of <0.05. Age was not correlated with current BMI, but the group who lost weight prior to PET scanning was nominally older than the group that gained weight: F = 4.46, *p* = 0.04 (35.0 ± 8.8 years vs. 30.6 ± 7.4 years). There were no age-by-BMI interaction effects for any of the PET measures. There was a nominal effect of sex on current BMI for the [^11^C]-NNC112 analysis (t = 2.03, *p* = 0.046), but none for the [^18^F]-FDOPA or [^18^F]-Fallypride scans, and no sex differences between the BMI-change groups for any of the PET scans. We controlled for age and sex in the ROI and voxelwise PET results reported below, which are listed in Tables 2 and 3.

### Current BMI is positively correlated with presynaptic dopamine synthesis capacity in the midbrain and hypothalamus and negatively correlated with D_2/3_ receptor BP_ND_ in the midbrain

**Fig. 1:**
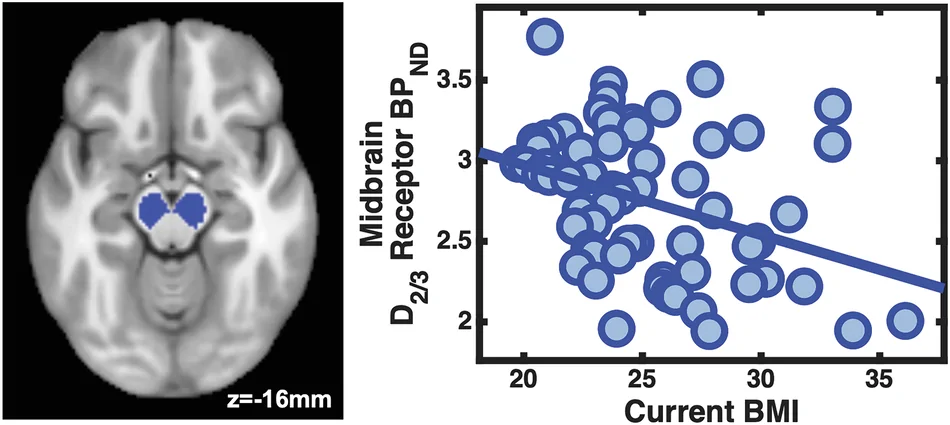
BMI correlates negatively with midbrain D_2/3_ receptor availability. Left: Example midbrain region of interest (ROI) from one representative participant overlaid on a structural MRI image. Right: Scatter plot showing the relationship between current BMI (*x*-axis) and D_2/3_ receptor binding potential (BP_ND_, *y*-axis) in the same ROI (*t* = −2.20, *p* = 0.032).

**Fig. 2:**
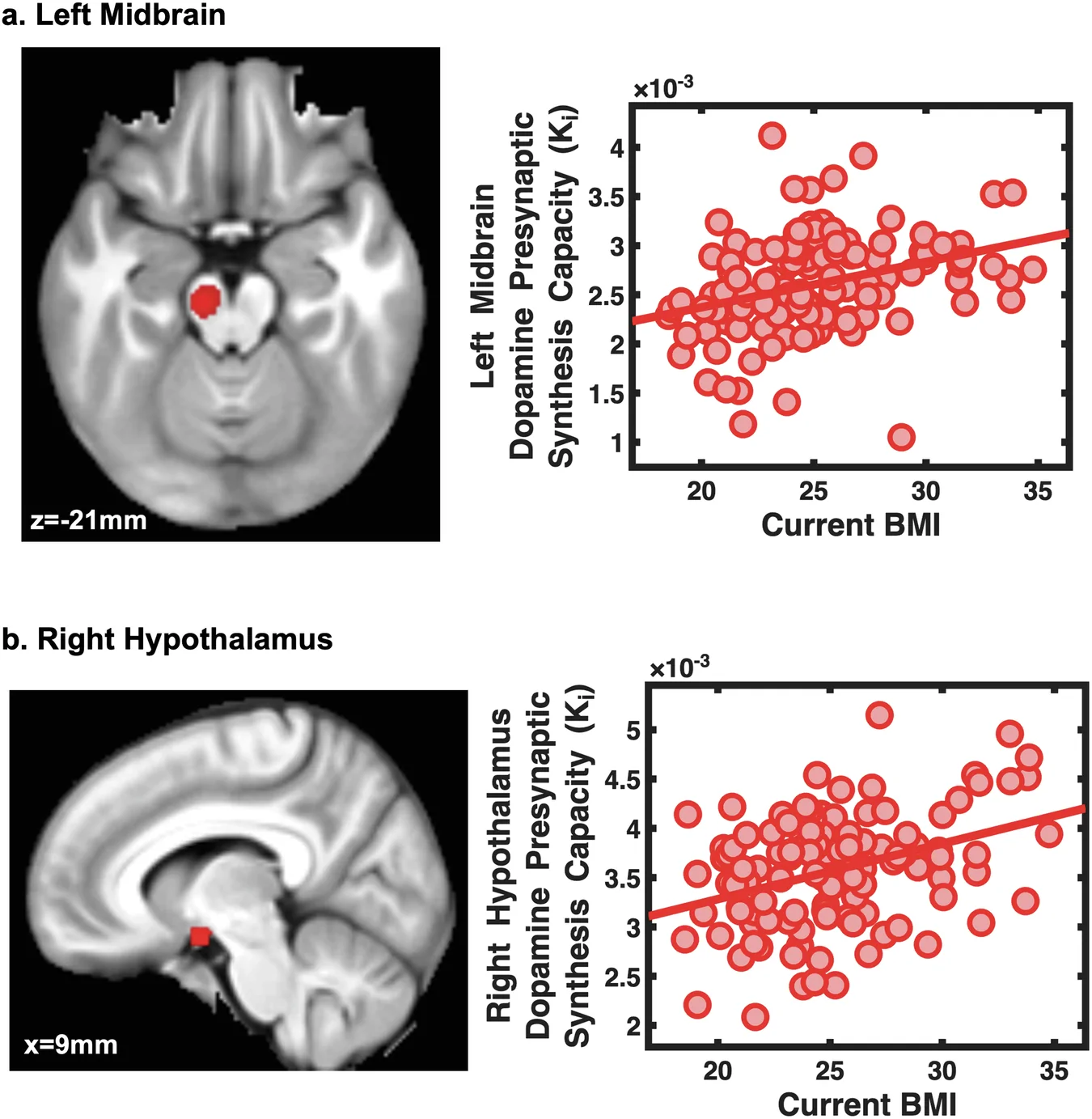
BMI correlates positively with dopamine synthesis capacity in the midbrain and hypothalamus. Voxelwise results showing the positive correlation between current BMI and dopamine synthesis capacity (*K*_i_) in **a** the left midbrain (peak voxel MNI coordinates: −10.5, −21, −21; *z* = 3.60, tfce-corrected *p*_FWE_ = 0.025) and **b** the right hypothalamus (peak voxel MNI coordinates: 9, 0, −16.5; *z* = 3.99, tfce-corrected *p*_FWE_ = 0.026). For each region, the significant cluster is overlaid on a structural MRI image (left), alongside a scatter plot showing the relationship between BMI and *K*_i_ extracted from the peak voxel (right). For visualization, images are shown at a *z*-stat threshold of 2.81.

We first investigated the dopaminergic basis of current BMI by testing for associations between current BMI and native-space dopamine PET ROI values for each of the three dopamine PET tracers (complete ROI results in Table 2). We found that BMI was negatively correlated with D_2/3_ receptor BP_ND_ in the midbrain (Fig. 1), but there were no correlations between current BMI and presynaptic synthesis capacity or D_1_ receptor BP_ND_ in any region of interest. Next, we performed voxelwise linear regression analyses for each PET tracer (restricted to a predefined mask including the basal ganglia and hypothalamus) to further localize effects. We found that current BMI correlated positively with presynaptic dopamine synthesis capacity in the left midbrain (Fig. 2a) and right hypothalamus (Fig. 2b). There were no significant correlations between current BMI and D_1_ or D_2/3_ receptor BP_ND_ in the voxelwise analysis.

### Prior BMI change is associated with mesolimbic presynaptic dopamine synthesis capacity

**Fig. 3:**
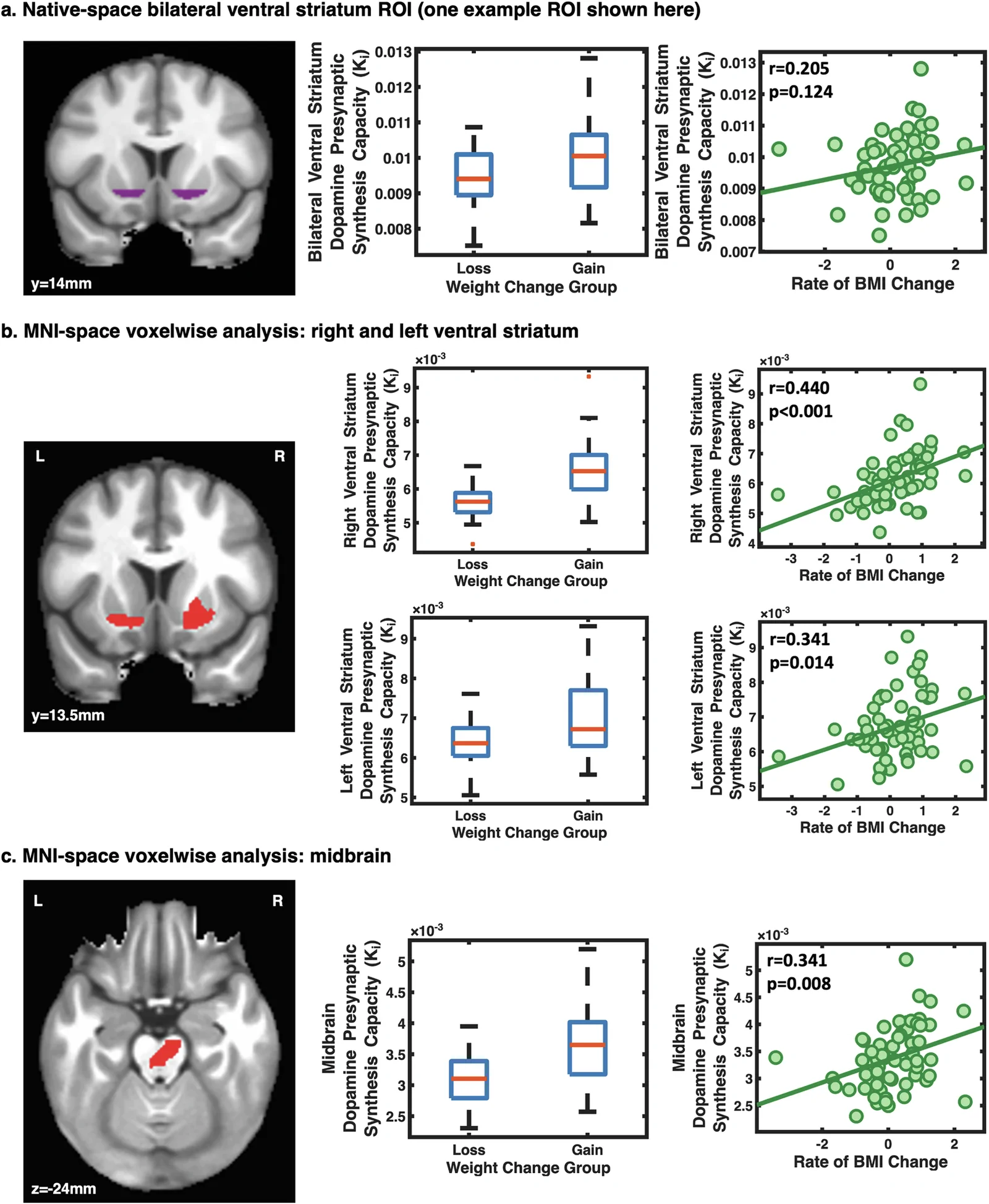
Weight gain is associated with increased dopamine synthesis capacity in the ventral striatum and midbrain. Dopamine synthesis capacity was higher in individuals who gained weight compared to those who lost weight prior to the PET scan, in the ventral striatum in **a** native-space ROI analysis (*F* = 6.89, *p* = 0.011) and **b** MNI-space voxelwise analyses: right ventral striatum (peak voxel MNI coordinates: 21, 6, −13.5, *z*-stat = 4.40, tfce-corrected *p*_FWE_ = 0.001) and left ventral striatum (peak voxel MNI coordinates: −21, 13.5, −12, *z*-stat = 3.12, tfce-corrected *p*_FWE_ = 0.028). **c** A corresponding effect was also observed in the midbrain in the voxelwise analysis (peak-voxel MNI coordinates: −1.5, −30, −24, *z*-stat = 3.92, tfce-corrected *p*_FWE_ = 0.015). For each region, panels show the ROI or significant cluster overlaid on a structural MRI (left), a boxplot comparing *K*_i_ values between the weight-gain and weight-loss groups (middle), and a scatter plot of the relationship between rate of BMI change and *K*_i_ (right, shown in green). This relationship was not significant for the bilateral ventral striatum ROI (*r* = 0.205, *p* = 0.124) but was significant for the peak voxels from the voxelwise analyses (right ventral striatum: *r* = 0.440, *p* < 0.001; left ventral striatum: *r* = 0.316, *p* = 0.014; midbrain: *r* = 0.341, *p* = 0.008). For visualization, voxelwise clusters are shown at a *z*-stat threshold of 2.81.

To investigate the dynamic relationship between BMI change and dopamine PET measures, we divided our cohort into those who gained (mean weight gain 3.67 ± 3.44 percent change in BMI) vs. those who lost weight (mean weight loss 3.07 ± 2.63 percent change in BMI) in the one to two years prior to the PET scans. We found that individuals who gained weight prior to the PET scan had increased dopamine synthesis capacity in the ventral striatum compared to those who lost weight (Fig. 3). This finding reached significance in both the native-space ROI analysis (Fig. 3a) and MNI space voxelwise analysis (Fig. 3b). In addition, in the voxelwise analysis, we observed increased presynaptic dopamine synthesis capacity in the left midbrain (Fig. 3c) for individuals who gained weight compared to those who lost weight. Importantly, these results were consistent in a post-hoc analysis employing the rate of prior BMI change as a continuous variable, which revealed a positive correlation between rate of prior BMI change and presynaptic dopamine synthesis capacity in the right ventral striatum, left ventral striatum, and midbrain (depicted in green on the right panels of Fig. 3a-c). We did not find associations between dopamine D_1_ or D_2/3_ receptor BP_ND_ and rate of prior BMI change.

## Discussion

In a sample of healthy humans with BMIs ranging from normal weight to moderately obese, we found that increased BMI was related to enhanced dopamine tone (i.e., presynaptic dopamine synthesis capacity) along with diminished dopamine receptor availability. Specifically, there was a positive correlation between current BMI and dopamine synthesis capacity in both the midbrain and hypothalamus, dopamine neuron rich regions that are important for reward processing and satiety regulation, respectively, but that have not been examined by prior PET studies of obesity. In concert, we observed lower D_2/3_ receptor availability in the midbrain, a region where D_2_ receptors play a key autoregulatory role in the presynaptic management of dopaminergic efflux in the striatum. Together, these data support a role for dopamine system function in body mass regulation and delineate specific pre- and post-synaptic elements of this role emanating from two distinct populations of dopaminergic neurons.

### Midbrain and hypothalamic dopamine synthesis capacity in relation to BMI

We observed a robust positive association between current BMI and dopamine synthesis capacity in the midbrain, but did not find such an effect in the striatum. The lack of a significant relationship between concurrent BMI and striatal presynaptic dopamine synthesis capacity in the present study may be due to methodological differences including our use of a different radiotracer, a larger sample size, or more constrained variation in the BMI range of participants compared to earlier work [34, 35, 54]. Given that midbrain [^18^F]-FDOPA specific uptake has been shown to positively correlate with reward-related responses in healthy young adults [55], the present finding—located in a region dense with dopamine-producing neurons involved in encoding reward expectation and prediction errors [12, 56]—aligns with the reward-surfeit model of obesity [40, 41], which posits heightened sensitivity to reward cues. However, longitudinal or mechanistic studies will be needed to directly test that interpretation.

Additionally, we found that current BMI positively correlated with presynaptic dopamine synthesis capacity in the hypothalamus, a region critical for the homeostatic regulation of appetite. Rodent studies provide valuable context for interpreting this relationship. Dopamine release in the lateral and ventromedial hypothalamus has been associated with increased meal size in normal-weight rats [57, 58], suggesting a role in satiety signaling. However, this mechanism may be reduced in obesity, as obese rats exhibit elevated basal hypothalamic dopamine levels and greater dopamine release during meal consumption compared to controls [59, 60]. These alterations in hypothalamic dopaminergic tone may have broader implications, extending beyond local neuroendocrine regulation. For example, electrical stimulation of the lateral hypothalamus increases dopamine release in the nucleus accumbens [61], enhancing appetite for palatable food and increasing meal size in rats [62]. While our human imaging data cannot resolve individual hypothalamic nuclei or establish causality, the observed positive relationship between BMI and hypothalamic dopamine synthesis capacity is consistent with preclinical evidence suggesting that elevated BMI may reflect disrupted dopaminergic satiety signaling in this region.

### Inverse association between BMI and D_2/3_ receptor binding potential

In line with independent past work [17, 18], we also found that current BMI correlated inversely with D_2/3_ receptor binding potential in the brains of human volunteers across the BMI spectrum, adding support to the link between mesolimbic dopamine systems and body mass regulation. We observed the most robust effect in the dopaminergic midbrain, an area where presynaptic autoreceptors abound, which has not been assessed in previous striatally-focused studies. Though the current design cannot distinguish with certainty between pre- and post-synaptic receptor binding, nor between dopamine D_2_ and D_3_ receptors, our results are concordant with previous findings that D_2_ autoreceptor deficient mice show increased food-seeking behavior [63]. Paired with the increased presynaptic dopamine synthesis capacity we observed in the midbrain of individuals with greater BMI, this result supports the hypothesis that lower autoreceptor availability (and greater local dopaminergic tone) in the midbrain could also be associated with disinhibited food behavior in humans as seen in murine models.

We cannot rule out the possibility that a portion of the variation in D_2/3_ receptor BP_ND_ may also reflect tracer competition by endogenous dopamine at the receptor site. In such a case, reduced D_2/3_ receptor BP_ND_ in individuals with higher BMI could reflect decreased receptor abundance and/or affinity, increased synaptic dopamine levels, or a combination of both. However, future experiments, such as those employing pharmacological dopamine depletion methods to estimate basal endogenous dopamine levels, would be needed to disentangle effects of receptor properties from those of endogenous dopamine competition.

Although not significant at the Bonferroni-corrected level, we also found a trend-level negative correlation between BMI and ventral striatum D_2/3_ receptor BP_ND_, consistent with past studies in humans [17] and rodents [18] that have championed reward deficiency theories of obesity. Because BMI status is a complex measure incorporating environmental, behavioral and metabolic factors, future work will be needed to understand the causal mechanisms behind these associations.

### Mesolimbic dopamine links to body mass dynamics

To better account for the dynamic nature of body mass regulation, we examined not only current BMI status effects, but also those of BMI trajectory. Notably, individuals who had recently gained weight showed significantly higher presynaptic dopamine synthesis capacity in the midbrain and ventral striatum—regions critically involved in reward processing—compared to those with recent weight loss. This difference remained significant even after statistically controlling for current BMI, indicating that the effect was independent of body mass at the time of assessment. Moreover, dopamine synthesis capacity in these key reward regions was positively associated with the rate of weight change, suggesting that mesolimbic dopamine function may play a critical role in both the magnitude and the temporal dynamics of recent weight change.

These observations support two potential interpretations: (1) individuals with elevated mesolimbic dopamine synthesis capacity may be more susceptible to accelerated weight gain, whereas those with decreased capacity are more prone to weight loss; or (2) recent weight change itself—either gain or loss—may induce corresponding alterations in mesolimbic dopamine tone. Longitudinal studies incorporating repeated PET imaging before and after weight change will be essential to disentangle these possibilities and to clarify the directionality of this relationship.

### Limitations

Although the present study provides insight into the dopaminergic correlates of body mass fluctuations, several limitations should be considered. First, the cross-sectional nature of the dopamine imaging limits our ability to draw causal inferences regarding dopamine-BMI associations. Future studies incorporating prospective BMI measurements alongside repeated dopamine imaging would help clarify their temporal dynamics. Additionally, weight changes in the 1–2 years preceding PET imaging were not available for all participants, which may have introduced variability that was not fully captured in our analyses; however, the subgroups included in the weight change analyses did not differ from the larger sample in age or sex distribution.

Although statistically robust, our results are not immune to the effects of measurement noise, especially in smaller regions, such as the dopaminergic midbrain, and replication work will be important. In addition, because PET measurements were collected cross-sectionally and without experimental intervention, we were unable to account for dynamic aspects of synaptic, endogenous dopamine that may be important contributors to body mass regulation. Future studies incorporating pharmacological challenge designs would be well positioned to provide additional insights in this vein.

Our sample did not include individuals with severe obesity, and therefore our findings may not generalize beyond the BMI range represented in the cohort. Because BMI does not distinguish between fat and lean mass or capture fat distribution, measures such as waist circumference or other indices of central adiposity may provide more precise characterization of metabolically relevant body composition in future studies.

Participants in this study experienced relatively modest weight changes, which may limit the applicability of our findings to more substantial weight fluctuations. Studies with longer follow-up periods or intervention-based designs that induce larger weight changes may provide deeper insight into the dopaminergic mechanisms underlying more pronounced alterations in body mass.

In addition, future studies that document eating behavior and that examine how dietary patterns of reward-related feeding behaviors may relate to dopaminergic measures are warranted. Metabolic factors, including insulin and other peripheral signals known to interact with dopaminergic function, were also not measured and may represent important mediators of the observed associations.

## Conclusion

Taken together, our multitracer PET study of a large sample of psychiatrically healthy controls revealed distinct alterations in presynaptic dopamine synthesis capacity and D_2/3_ receptor availability in key reward and homeostatic regions associated with increased BMI. Longitudinal BMI assessments further demonstrated that mesolimbic dopamine synthesis capacity is closely linked not only to weight change but also to the rate of that change. These results underscore the need for extended longitudinal studies to clarify the dynamic role of dopaminergic dysfunction in obesity and may ultimately inform the development of targeted interventions.

## Supplementary information

Supplemental Material

## Data Availability

Summary data from this study are available upon reasonable request. All data sharing is subject to compliance with individual participant consent forms and, where applicable, review by the NIH Institutional Review Board.
