# Supplementary material for: Body mass regulation in humans is associated with dopamine synthesis capacity and dopamine receptor profile measured with positron emission tomography
Source: Transl Psychiatry. 2026 Aug 13;16:636. doi: 10.1038/s41398-026-04318-6 (PMC13759531; doi:10.1038/s41398-026-04318-6)
Supplement: Supplementary file 1 [file 41398_2026_4318_MOESM1_ESM.docx]

**Supplemental material**

*Ianni et al, 2026.*

**eFigure 1. Cross-correlation matrix of PET ROI values across tracers.** Heatmap showing pairwise Pearson correlations among region-of-interest (ROI) PET measures across tracers: [^18^F]-FDOPA (FDOPA), [^18^F]-Fallypride (FALLY), and [^11^C]-NNC112 (NNC). Values reflect inter-regional and inter-tracer associations in standardized uptake/availability estimates, with warmer colors indicating stronger positive correlations and cooler colors indicating negative correlations. Correlations are shown for all ROI pairs included in the analysis; lower triangle masking is applied for visualization clarity.

**eTable 1. Descriptive statistics for PET imaging outcome measures by tracer and region of interest.** Summary statistics are presented for three PET tracers: [18F]-FDOPA, [18F]-Fallypride, and [11C]-NNC112. Regions of interest include the caudate nucleus, putamen, ventral striatum, and midbrain (the latter not available for [11C]-NNC112). Mean, standard deviation (SD), minimum, and maximum are reported for each tracer-ROI combination. K_i_ values are multiplied by 10^2^ for display purposes.
